# Longitudinal Clinical Progression in X‐Linked Adrenoleukodystrophy: The AMNL Scoring System

**DOI:** 10.1002/cns3.70084

**Published:** 2026-08-04

**Authors:** Eda G. Kabak, Cecilie Videbaek, Marije M. C. Voermans, Björn M. van Geel, Keith van Haren, Stephan Kemp, Diederik van de Beek, Marc Engelen

**Affiliations:** ^1^ Departments of Neurology and Pediatric Neurology, Emma Children's Hospital, Amsterdam Leukodystrophy Center, Amsterdam University Medical Center, Amsterdam Neuroscience University of Amsterdam Amsterdam the Netherlands; ^2^ Department of Neurology Stanford University School of Medicine Palo Alto California USA; ^3^ Department of Pediatrics and Clinical Genetics Centre for Inherited Metabolic Diseases, Copenhagen University Hospital, Rigshospitalet Copenhagen Denmark; ^4^ Noordwest Ziekenhuisgroep Alkmaar the Netherlands; ^5^ Laboratory Genetic Metabolic Diseases, Department of Laboratory Medicine, Amsterdam University Medical Center, Amsterdam Gastroenterology Endocrinology Metabolism University of Amsterdam Amsterdam the Netherlands; ^6^ Department of Neurology, Amsterdam University Medical Center, University of Amsterdam Amsterdam Neuroscience Amsterdam the Netherlands

**Keywords:** ABCD‐1 deficiency, ALD, AMNL score, leukodystrophy, myelopathy

## Abstract

**Objective:**

The current clinical nomenclature for individuals with *ABCD1* gene dysfunction is often uninformative. The disorder was initially described as a combination of adrenal insufficiency and leukodystrophy, leading to the widespread use of “X‐linked adrenoleukodystrophy” (ALD). However, this term is inaccurate for individuals who never develop these features. *ABCD1* dysfunction can cause various symptom complexes: adrenal insufficiency, myelopathy, neuropathy, and leukodystrophy, which may occur consecutively, simultaneously, or not at all.

**Methods:**

We developed an intuitive annotation system to precisely convey phenotype status for individuals with *ABCD1* dysfunction. The “AMNL score” assesses the presence and severity of [A]drenal insufficiency, [M]yelopathy, [N]europathy, and [L]eukodystrophy, with scores ranging from 0 (no symptoms) to 3 (severe) per domain.

**Results:**

Applied to initial clinical encounters with 101 Dutch patients and 30 patients from the California newborn screening (NBS) program, the system identified 33 unique presentations. The most common was isolated mild myelopathy (A0M1N0L0). Including predominantly asymptomatic NBS newborns (mostly A0M0NUL0) broadened the spectrum and highlighted the need for precise classification across ages. Longitudinal use over 2 years in 99 Dutch patients showed progression in one or more domains in 41.4%, most often worsening myelopathy. In the NBS cohort (median follow‐up: 3.5 years; range: 0.3–12.2), most patients showed no myelopathy or leukodystrophy, though adrenal involvement was detected in half. Initial inter‐rater reliability was high (*α* = 0.996).

**Interpretation:**

The AMNL score's ability to quantify domain‐specific changes over time makes it valuable for monitoring disease evolution in clinical practice and research.

## 1 Introduction

X‐linked adrenoleukodystrophy (ALD), the most common peroxisomal disorder, is caused by pathogenic variants in the *ABCD1* gene. *ABCD1* deficiency results in impaired degradation and accumulation of very long‐chain fatty acids (VLCFAs). First described in the late 19th century [1], long before its genetic and biochemical cause was identified, several white matter disorders were described based on histopathology. Terms such as “diffuse hirnsclerose” and “Schilder's disease” were used. These were broad pre‐etiological umbrella terms that encompassed a variety of genetic and potentially acquired white matter disorders, including ALD [2, 3, 4, 5]. The term “adrenoleukodystrophy” introduced by Blaw in the 1970s is still in use today [6]. Before biochemical and genetic testing was available, the diagnosis relied on the classic clinical presentation (rapid neurological decline and primary adrenal failure in male children) and characteristic postmortem findings on autopsy [7]. However, this changed when pathognomonic trilamellar inclusions were discovered in postmortem brain, adrenal, and testicular tissue from patients with ALD using electron microscopy [8, 9] and especially when it was shown that VLCFAs are elevated in plasma [10]. The availability of a diagnostic biomarker, total C26:0 and the C26:0/C22:0 ratio in plasma, was a game changer that broadened the clinical spectrum. A large group of male patients with progressive spinal cord disease, peripheral neuropathy, and often adrenal failure, who were classified as having “hereditary spastic paraparesis,” were shown to have increased VLCFA in plasma and therefore also ALD, albeit with a completely different presentation. These patients were referred to as having “adrenomyeloneuropathy” (AMN) and were initially considered to have a distinct adult presentation [9, 11]. In 1993, the *ABCD1* gene was identified as the causative gene, making genetic testing possible [12].

This further expanded the ALD spectrum, and our understanding of its natural history evolved from “static phenotypes” (e.g., “cerebral ALD,” “Addison only,” “adrenomyeloneuropathy”) to a progressive neurodegenerative disorder with a highly variable presentation where symptoms accumulate over time [13]. Our current understanding of the natural history of ALD shows that all patients are asymptomatic at birth. In male patients, adrenal failure is common, affecting 60% before the age of 10, with a lifetime prevalence of 85%. Spinal cord disease occurs in adulthood, with a lifetime prevalence of probably close to 100% [14, 15]. Leukodystrophy can occur in male patients, with the earliest cases appearing around 2 years of age and a lifetime prevalence of 60% [16]. The combination of the different clinical syndromes and age of onset is variable. In women, adrenal failure and leukodystrophy are very rare, but spinal cord disease affects the vast majority eventually (around 90% of women over 60 years of age) [17, 18].

Over the decades, a higher rate of diagnosis has led to a complex web of “phenotypes,” including “cerebral ALD,” “AMN,” “Addison‐only,” and “overlap” forms [19]. Despite natural history studies showing that *ABCD1* deficiency is a progressive neurometabolic disorder in which symptoms accumulate over a lifetime [14, 13], this static phenotypic categorization is still widely used. This outdated nomenclature leads to convoluted descriptions and misconceptions about the disease's natural history. This problem is exacerbated by the identification of completely asymptomatic individuals through neonatal screening (NBS).

We developed the AMNL score, a clinical rating scale that integrates the core clinical features of *ABCD1* deficiency: adrenal insufficiency (A), myelopathy (M), neuropathy (N), and leukodystrophy (L). Here, we report the first application and validation of the AMNL score in a cohort of patients with *ABCD1* deficiency. We assessed the usability and inter‐rater reliability of the AMNL score. We also evaluated its ability to capture neurological progression over time. We propose using the AMNL as a classification system rather than a continuous outcome measure. The AMNL's primary purpose is to standardize communication between physicians and enable homogeneous patient selection for trials and retrospective clinical studies. It is an effective way to quickly capture disease severity in individuals with *ABCD1* deficiency.

## 2 Subjects and Methods

### 2.1 Study Population

We evaluated 101 individuals with confirmed pathogenic *ABCD1* variants who were enrolled in the Dutch ALD cohort and are being followed at the Amsterdam University Medical Center (UMC). The study included patients who were followed both retrospectively and prospectively. In addition to patients identified based on family history or clinical symptoms, we included 59 newborns identified through the California NBS program for ALD. Patients with a variant of uncertain significance (VUS) were excluded. This addition broadened the phenotypic spectrum and permitted evaluation across the complete disease course.

Clinical, biochemical, and radiological features were recorded during the initial evaluation at the Amsterdam UMC, a tertiary center specializing in ALD, and at Stanford Medicine. Patients in the Amsterdam UMC cohort are followed yearly as part of an ongoing natural history study [20] and are examined by an expert neurologist or physician‐researchers, who perform standardized measurements. Patient records are stored in both the local electronic patient reporting system as well as Castor EDC. For the purpose of the new scoring system, patient records were obtained retrospectively for the baseline values and after a 2‐year follow‐up period. The California NBS cohort is a sample of ALD patients referred to Stanford Medicine, a specialized ALD center that manages ALD care. All patients underwent a routine early evaluation of adrenal function in accordance with international ALD management guidelines [21]. For Stanford Medicine patients, data from the most recent visit were analyzed due to variation in longitudinal follow‐up.

Data collection for both cohorts was approved and conducted in accordance with institutional review boards (Stanford IRB #59014; AMC IRB 2018_310).

### 2.2 Development of the Adrenal Insufficiency, Myelopathy, Neuropathy, and Leukodystrophy (AMNL) Score

We designed a concise ordinal score that incorporates the four hallmark features of *ABCD1* deficiency: adrenal insufficiency, myelopathy, peripheral neuropathy, and leukodystrophy. Each domain was scored independently based on objective tests or clinically validated surrogates. When diagnostic information was unavailable, we assigned domain‐specific “unknown” values to maintain transparency. Over the years, two neurologists with ALD expertise (B.v.G. and M.E.) discussed the usefulness of a practical ALD scoring system, possibly based on the tumor, node, and metastasis (TNM) staging score used in oncology. An initial version of the score was created and refined by a physician researcher working on her PhD thesis (E.G.K.). Colleagues abroad were consulted to arrive at the current version. Table 1 shows the systematized scoring system.

**Table 1:** The systematic AMNL scoring system for the four hallmark features of *ABCD1* deficiency.

	Score				
Adrenal insufficiency	U	A0	A1	A2	A3
	Unknown	None	Stress dose glucocorticoid only	Glucocorticoid supplementation	Glucocorticoid and mineralocorticoid supplementation
Myelopathy	U	M0	M1	M2	M3
	Unknown	None	Signs and symptoms only	Motor disability but ambulatory	Wheelchair dependent
Neuropathy	U	N0	N1	N2	
	Unknown	None	Peripheral neuropathy of the lower extremities	Peripheral neuropathy of the upper and lower extremities	
Leukodystrophy	U	L0	L1	L2	L1*
	Unknown	None	Arrested CALD (no gadolinium enhancement)	Active CALD (gadolinium enhancement)	Status after HSCT therapy

#### 2.2.1 A–Adrenal Insufficiency

Adrenal function was classified from A0 to A3 based on treatment requirements, reflecting the severity of hypothalamic‐pituitary‐adrenal axis failure.

− **A0**: absence of adrenal insufficiency
− **A1**: stress‐dose glucocorticoids required (intermittent therapy)
− **A2**: daily glucocorticoid supplementation
− **A3**: combined glucocorticoid and mineralocorticoid supplementation required
− **U**: adrenal function unknown

The initiation of therapy depends on clinical features and/or the results from an ACTH (adrenocorticotropic hormone) stimulation test, plasma electrolytes, and plasma renin activity [14]. Scoring reflects the degree of hormonal axis failure, with A3 representing the most severe involvement.

#### 2.2.2 M–Myelopathy

Myelopathy was graded (M0–M3) depending on the degree of gait impairment and need for assistive devices, which reliably indicate progression of corticospinal and dorsal column pathology.

− **M0:** no signs of myelopathy
− **M1:** clinical myelopathy with independent ambulation
− **M2:** requires unilateral or bilateral walking aids
− **M3:** wheelchair dependence
− **MU:** neurological examination absent or gait function unknown

We used gait function as a reflection of myelopathy, as natural history studies demonstrate that a progressive gait disorder is the main symptom [22, 23] caused by degeneration of the dorsal columns and corticospinal tracts. As degeneration advances, patients increasingly rely on walking aids [24]. Thus, M1 is used for signs and symptoms with preserved independent walking, M2 for disability requiring unilateral or bilateral walking assistance, and M3 for wheelchair dependence. This simple metric of walking aid dependency was chosen for its ease of use and ability to be quickly scored, particularly with (limited) retrospective data. While the Expanded Disability Status Scale (EDSS) offers a more detailed assessment of walking function, its non‐linear nature and more complex scoring system may make it less intuitive and harder to apply in routine or rapid assessments [24]. M‐U is used when the neurological examination is absent or gait function is unknown. M3 is assigned only when a patient is essentially fully wheelchair dependent and can take no more than a few steps independently. If a patient can walk more than a few steps despite primarily using a wheelchair, an M2 score is assigned.

#### 2.2.3 N–Neuropathy

Peripheral neuropathy can only be scored if neurophysiological testing was done (sensory nerve action potentials, compound muscle action potentials, and conduction velocities) and was scored according to anatomical extent.

− **N0:** no neuropathy
− **N1:** neuropathy only in the lower extremities
− **N2:** neuropathy in both upper and lower extremities
− **NU:** electrodiagnostic examination is absent

This is because assessing peripheral nerve involvement by history and neurological examination alone is difficult due to the prominent signs of spinal cord disease (like absent vibration sense in the lower extremities due to dorsal column dysfunction) in most patients [25]. Severity is graded by whether the neuropathy is localized in the lower extremities only or if the upper extremities are also involved. The neurophysiological features of the neuropathy were not specified because there is no consensus in the literature on whether the neuropathy is primarily axonal or demyelinating [26, 27], and histological studies are not conclusive [11, 28, 29].

#### 2.2.4 L–Leukodystrophy

This element describes the presence and degree of cerebral leukodystrophy. L0 indicates the absence of ALD‐related white matter lesions. The severity is characterized by the presence or absence of gadolinium enhancement on MRI, a finding that suggests progressive disease [30]. Accordingly, L1 describes arrested/stable leukodystrophy ALD (no gadolinium enhancement), while L2 describes progressive leukodystrophy (with gadolinium enhancement). Because hematopoietic stem cell therapy (HSCT) is the sole available therapy to arrest demyelination [30], patients who have undergone HSCT are classified separately as L1*. L‐U is used when MRI is not available.

### 2.3 Inter‐Rater Reliability

The inter‐rater reliability of the AMNL score was assessed in the Amsterdam UMC cohort. Scoring was performed independently by a physician‐researcher (E.G.K.) and a neurologist (M.E.), who were both blinded to each other's scores. Reliability was evaluated at the domain level; each patient contributed four independently scored domains. Discrepancies were resolved through discussion. Inter‐rater reliability was quantified using Cronbach *α*, which was calculated via the Spearman–Brown formula for two raters.

## 3 Results

### 3.1 Patient Characteristics

A total of 101 individuals with confirmed pathogenic *ABCD1* variants were included, together with 30 patients from the NBS cohort at Stanford Medicine. This group included patients with pathogenic *ABCD1* variants (*n* = 21) and likely pathogenic variants (*n* = 9). Individuals with a VUS (*n* = 29) were excluded from analyses. The Dutch cohort had a median age of 30.5 years (range 12.0–69.0). All patients were scored with the proposed AMNL system at their baseline visit. In contrast, all patients in the newborn cohort were evaluated shortly after birth and had no clinical symptoms prior to enrollment.

### 3.2 Distributions of AMNL Scores at Baseline

**Figure 1:**
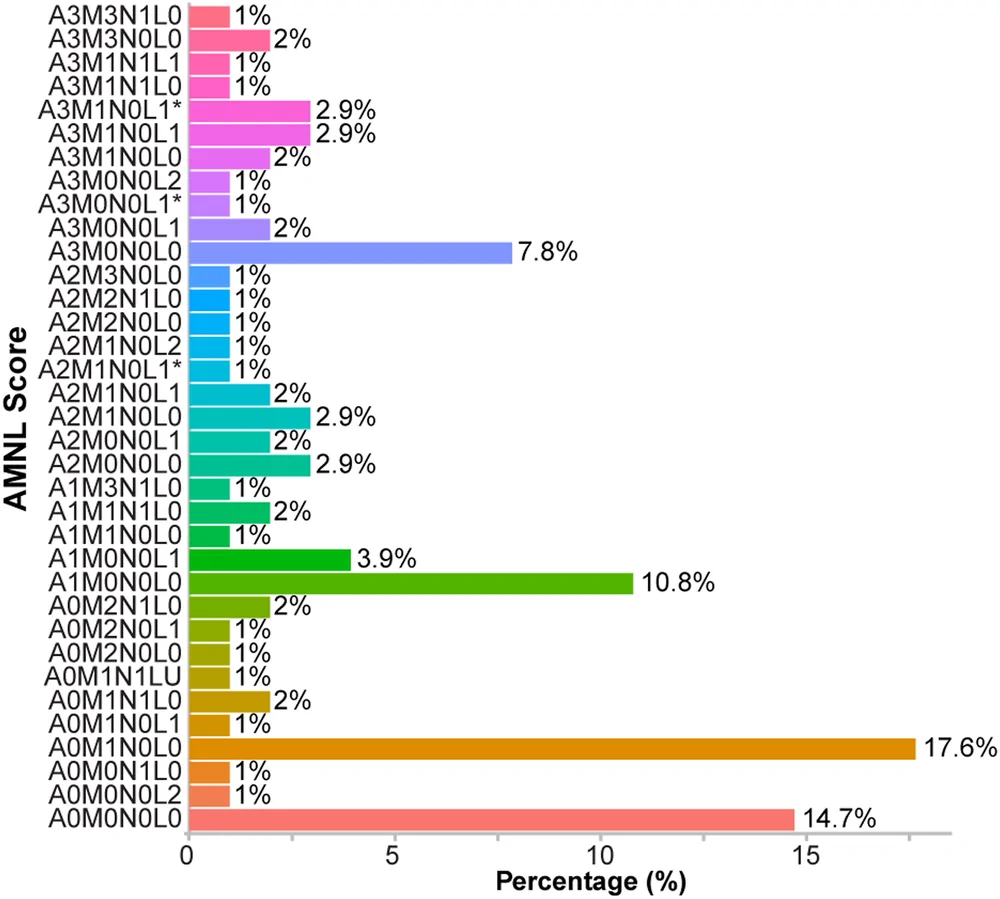
Distribution of AMNL (adrenal insufficiency, myelopathy, neuropathy, leukodystrophy) scores at baseline visit in male patients of the Dutch adrenoleukodystrophy (ALD) cohort.

Within the total group of 101 patients in the Dutch cohort, 33 different, unique AMNL score combinations were present at the baseline visit, highlighting the extensive clinical heterogeneity. The distribution of the most frequent AMNL scores is detailed in Figure 1.

The most common clinical presentation was an isolated myelopathy with minor symptomatology (A0M1N0L0), observed in 18 patients (17.6%). Asymptomatic individuals (A0M0N0L0) constituted 15 patients (14.7%). Isolated adrenal insufficiency (A1M0N0L0, A2M0N0L0, A3M0N0L0) was the presenting feature in 22 patients (21.5%).

### 3.3 Longitudinal AMNL Changes

**Figure 2:**
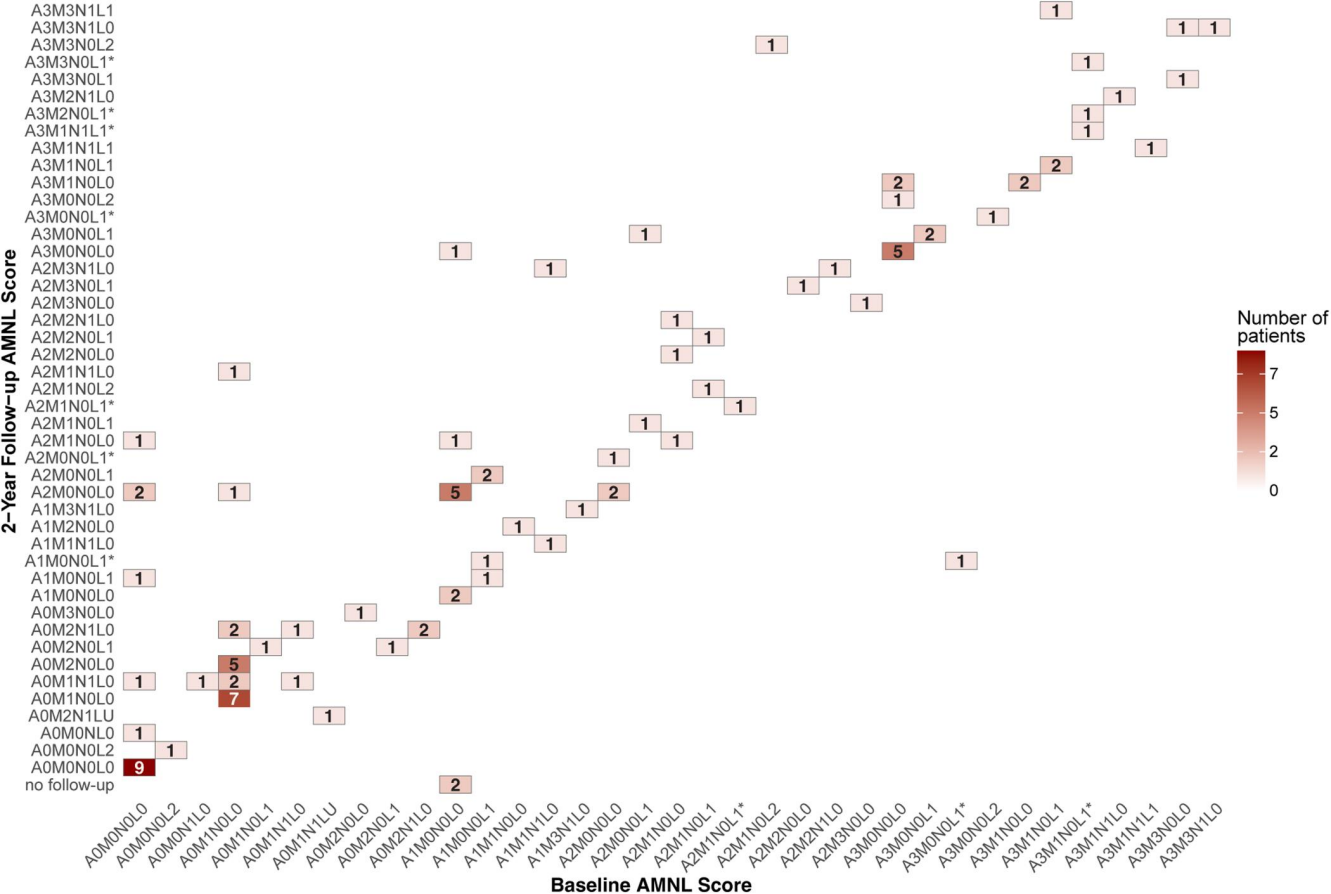
AMNL (adrenal insufficiency, myelopathy, neuropathy, leukodystrophy) score transition between baseline (X‐axis) and 2‐year follow‐up (Y‐axis). Each cell shows the number of patients with that specific transition.

Longitudinal AMNL score data were available for 99 patients. The transitions in AMNL scores between baseline and 2‐year follow‐up for the Dutch cohort are detailed in Figure 2. Over the 2‐year follow‐up period, disease burden was stable in 46 patients (46.5%), exhibiting no change in their AMNL score. Among the 18 patients who were completely asymptomatic (A0M0N0L0) at baseline, 11 (61.1%) remained asymptomatic at follow‐up. Disease progression, defined as an increase in any AMNL domain score, was observed in 41 patients (41.4%). The most frequent type of progression was worsening myelopathy (M‐score), which occurred in 23 patients. Five of the 18 initially asymptomatic patients (28%) developed new adrenal insufficiency during the follow‐up period. Seven patients in the full Dutch cohort, seven patients in total, had undergone HSCT and were classified as L1* (see Figure 1).

### 3.4 The NBS Subgroup

As expected, all 30 patients in the NBS cohort were asymptomatic at birth and scored as A0M0NUL0. The median age at the follow‐up visit was 3.5 years (range 0.3–12.2). At the follow‐up visit, the cohort's AMNL scores shifted, though most patients remained without adrenal involvement. The distribution of scores is detailed in Table 2. A0M0NUL0 remained the most common score, observed in 15 patients (50.0%), though any degree of adrenal involvement was detected in the other half of the cohort. Five patients had subclinical adrenal insufficiency (16.7%), and 10 required corticosteroid supplementation (33.3%). Two patients had arrested cerebral ALD. These results demonstrate the utility of the AMNL score in capturing the predominantly presymptomatic status of the NBS‐identified population at baseline and its ability to precisely document the onset of adrenal insufficiency during follow‐up.

**Table 2:** AMNL scores at follow‐up visit in male patients of the California NBS cohort.

	Frequencies
**A0M0NUL0**	15 (50.0%)
**A1M0NUL0**	5 (16.7%)
**A2M0NUL0**	8 (26.7%)
A2M0NUL1*	2 (6.7%)

### 3.5 Inter‐Rater Reliability

Across the 800 domain‐level observations (4 domains × 200 scoring occasions), there were seven mismatches, all of which occurred at the M2/M3 boundary of the M‐domain. This yielded an overall domain‐level matching rate of 99.1% (*α* = 0.996). The mismatches centered on patients who were predominantly wheelchair dependent but retained minimal ambulatory ability. This distinction is now clarified in the scoring criteria.

## 4 Discussion

We developed and applied the simple, practical AMNL score to describe the clinical status of individuals with *ABCD1* dysfunction. Our results, derived from a Dutch cohort of 101 patients and 30 patients from the Californian NBS cohort, demonstrate the system's ability to capture the remarkable heterogeneity of ALD at presentation. The system identified 33 distinct clinical presentations across the entire disease spectrum. Including NBS‐identified newborns further validates the score's relevance across the entire spectrum, from presymptomatic children to severely affected adults.

The current literature is inconsistent in the terminology used to describe affected patients. As a recent study highlights [31], studies vary in how they categorize patients with leukodystrophy (e.g., “adult‐onset cerebral ALD” or “occipital cerebral ALD”), and it is often not clear whether these patients with leukodystrophy have other disease features [26, 32, 33, 34, 35]. This inconsistency in terminology and lack of detail complicates the synthesis of natural history data and the comparison of outcomes across retrospective studies, which is essential in rare diseases. The AMNL score addresses these critical shortcomings of the historical phenotypic classification in ALD. Terms such as “AMN” or “Addison‐only” are static and fail to convey the multifaceted and progressive nature of the disease. An AMNL score of A2M1N0L0 immediately and concisely provides information about a patient. It indicates that the patient has glucocorticoid‐dependent adrenal insufficiency (A2) and mild signs of myelopathy (M1) but preserved independent gait. Additionally, the patient does not have peripheral neuropathy (N0) or cerebral leukodystrophy (L0). This level of detail establishes a common language for clinicians and researchers.

The AMNL score easily defines homogenous patient subgroups for trials and is useful for multicenter retrospective studies and long‐term follow‐up. It also facilitates clearer communication within multidisciplinary teams. The AMNL score establishes a common language for clinical care, improves prognostic counseling, and enables crucial patient stratification for evaluating new therapies in ALD clinical trials.

The AMNL score's utility extends beyond cross‐sectional classification, becoming a dynamic tool for tracking disease evolution. This is a significant improvement over traditional frameworks that rely on isolated measures, such as the EDSS [36] and SSPROM [37], which mainly score the myelopathy. Our longitudinal data showed that the AMNL score depicts multifaceted disease changes. This allows clinicians and researchers to transition from merely noting that a patient has “progressed” to specifying the nature of their progression. For example, it can differentiate isolated adrenal onset (A0 → A1) from progressive myelopathy (M1 → M2).

We acknowledge that the current score does not incorporate all potential manifestations. Testicular involvement, while histologically documented, is not a prominent clinical feature and was therefore excluded. Cognitive impairment, which is often associated with cerebral leukodystrophy, is indirectly captured by the “L” score. As our understanding of the disease evolves, future refinements to the score could be needed. Another limitation of this work is that the AMNL score is in the early stages of formal validation. Although inter‐rater reliability showed an excellent rate (Cronbach *α* = 0.996), broader validation is needed before the score can be fully endorsed for these purposes. This includes prospective, multicenter application and assessment by raters with varying levels of ALD expertise.

Additionally, we reiterate the argument that “*ABCD1* deficiency syndrome” is a more accurate term than “adrenoleukodystrophy” because many patients never develop leukodystrophy or adrenal failure. Although changing established terminology is challenging, the advent of NBS makes this discussion more urgent than ever. It is likely that the disease spectrum will expand further. We are now identifying individuals with mild biochemical abnormalities that are not seen in individuals with a classic disease presentation. The disease course in these individuals will likely be different, with (very) late onset of mild spinal cord disease and peripheral neuropathy, and a very low risk of adrenal failure and leukodystrophy [38]. Current risk estimates, such as the lifetime prevalence of leukodystrophy at 60%, are likely too high. Therefore, the term "adrenoleukodystrophy" is likely inappropriate for many individuals identified through NBS. Combining a more accurate disease name with the descriptive AMNL score would provide optimal clarity.

## 5 Conclusion

The AMNL score facilitates communication between healthcare providers and conveys concise information on the clinical status of individuals with adrenoleukodystrophy, from those identified presymptomatically via NBS to those with advanced multisystem involvement. Adopting the AMNL score in clinical practice and research will improve patient management and facilitate natural history studies. In line with modern practice, it is also appropriate to replace the diagnosis of adrenoleukodystrophy with the diagnosis of *ABCD1* deficiency syndrome.

## Author Contributions

**Eda G. Kabak:** writing – original draft, visualization, data curation, methodology, investigation. **Cecilie Videbaek:** writing—review and editing. **Marije M. C. Voermans:** data curation. **Björn M. van Geel:** writing – review and editing. **Keith van Haren:** writing – review and editing, conceptualization. **Stephan Kemp:** writing – review and editing, visualization. **Diederik van de Beek:** writing – review and editing. **Marc Engelen:** conceptualization, methodology, writing – review and editing, investigation.

## Ethics Statement

All procedures followed were in accordance with the ethical standards of the responsible committee on human experimentation (institutional and national) and with the Helsinki Declaration of 1975, as revised in 2000. Informed consent was obtained from all patients for being included in the study. Ethical approval for this study was given by the local medical ethical review committee of the Amsterdam UMC.

## Consent

For the Dutch cohort: Informed consent was gathered from all included patients and is available from the corresponding author. For the NBS cohort: Data were collected under the IRB 59014, which allows for the collection of retrospective data from medical charts.

## Conflicts of Interest

Cecilie Videbaek has received travel support from Sanofi. Keith van Haren has received clinical trial funding from Minoryx, IONIS, bluebird bio, and the National Institutes of Health; has received research support from Minoryx; and serves on a Data Safety and Monitoring Committee for Calico/AbbVie. Marc Engelen has received research support from Minoryx, Autobahn Therapeutics, bluebird bio, and SwanBio; consultation fees from Minoryx, bluebird bio, Autobahn Therapeutics, Poxel, Spolia, and Mitsubishi Tanabe. All other authors declare no conflicts of interest.

## Data Availability

The data that support the findings of this study are available from the corresponding author upon reasonable request.
